# Plasma C1q/TNF-Related Protein-9 Levels Are Associated with Atherosclerosis in Patients with Type 2 Diabetes without Renal Dysfunction

**DOI:** 10.1155/2016/8624313

**Published:** 2016-12-14

**Authors:** Mariko Asada, Tomoaki Morioka, Yuko Yamazaki, Yoshinori Kakutani, Reina Kawarabayashi, Koka Motoyama, Katsuhito Mori, Shinya Fukumoto, Atsushi Shioi, Tetsuo Shoji, Masanori Emoto, Masaaki Inaba

**Affiliations:** ^1^Department of Metabolism, Endocrinology and Molecular Medicine, Osaka City University Graduate School of Medicine, 1-4-3, Asahi-machi, Abeno-ku, Osaka 545-8585, Japan; ^2^Department of Premier Preventive Medicine, Osaka City University Graduate School of Medicine, 1-4-3, Asahi-machi, Abeno-ku, Osaka 545-8585, Japan; ^3^Department of Vascular Medicine, Osaka City University Graduate School of Medicine, 1-4-3, Asahi-machi, Abeno-ku, Osaka 545-8585, Japan

## Abstract

*Aim.* C1q/tumor necrosis factor-related protein-9 (CTRP9), a paralog of adiponectin, is expressed in adipose tissue. CTRP9 exerts protective effects against obesity and atherosclerosis in rodents. We investigated the association between plasma CTRP9 levels and atherosclerosis in patients with type 2 diabetes.* Methods.* We included 419 patients with type 2 diabetes, 161 of whom had chronic kidney disease (CKD). Fasting plasma CTRP9 and total adiponectin levels were measured with enzyme-linked immunosorbent assay. The intima-media thickness (IMT) of the common carotid artery was measured with ultrasonography.* Results*. Plasma CTRP9 levels were higher in the CKD group than in the non-CKD group. Plasma CTRP9 levels were positively correlated with carotid IMT in the non-CKD group. Multivariate analyses revealed that plasma CTRP9 levels were positively associated with carotid IMT in the non-CKD group, independent of age, sex, body mass index, adiponectin, and other cardiovascular risk factors. However, plasma CTRP9 levels were not associated with carotid IMT in the CKD group.* Conclusion.* Plasma CTRP9 levels are associated with atherosclerosis in diabetic patients without CKD, independently of obesity, adiponectin, and traditional cardiovascular risk factors. This study indicates a potential role of CTRP9 in atherosclerosis progression in human type 2 diabetes.

## 1. Introduction

Adipose tissue exerts endocrine and immune functions by releasing bioactive mediators termed adipokines [1]. Recent evidence has shown that fat accumulation, especially in the abdominal cavity, causes dysregulation of adipokines, including increase in leptin, tumor necrosis factor- (TNF-) *α*, interleukin-6, and monocyte chemotactic protein-1, and decrease in adiponectin, leading to the development of various metabolic disorders and atherosclerotic cardiovascular diseases [1, 2]. Adiponectin, a member of the C1q/TNF-related protein (CTRP) family, is one of the most extensively studied adipokines that possesses insulin-sensitizing, anti-inflammatory and antiatherogenic effects [3–5]. To date, 15 additional CTRP family members have been identified that are related to adiponectin in sequence and structural organization [6, 7].

Of all the CTRPs, CTRP9 has the highest amino acid identity to adiponectin in its globular C1q domain [6, 8]. CTRP9 is predominantly expressed in the adipose tissue and plays protective roles in diet-induced obesity, glucose intolerance, and insulin resistance in mice [8, 9]. Several basic studies have shown the beneficial effects of CTRP9 on the cardiovascular system [10–14]. CTRP9 was shown to induce endothelium-dependent vasorelaxation [14], attenuate neointimal formation after vascular injury [13], and protect against cardiac injury [10, 11], and adverse cardiac remodeling [12] after acute infarction in mice. Consistent with those experimental studies, serum CTRP9 levels were found to be inversely related to obesity, insulin resistance, and dyslipidemia in a community-based population [15] and in patients with coronary artery disease [16]. In contrast, serum CTRP9 levels were positively associated with BMI in morbidly obese subjects requiring bariatric surgery [17] and with obesity, insulin resistance, and arterial stiffness in subjects with type 2 diabetes (T2D) [18].

No study is currently available on the association between CTRP9 and morphological evidence of atherosclerosis in human subjects. Moreover, the association of CTRP9 with atherosclerosis remains to be fully investigated because patients with renal dysfunction or chronic kidney disease (CKD), who have a high risk of cardiovascular mortality [19], were not included in the preceding studies [15, 16, 18]. Therefore, in this study, we measured the plasma levels of CTRP9 in subjects with T2D representing a wide range of renal function and investigated the clinical association of plasma CTRP9 level with the intima-media thickness (IMT) of the carotid artery separately in subjects with CKD and in those without CKD.

## 2. Materials and Methods

### 2.1. Subjects

We consecutively enrolled 419 subjects with T2D (245 men and 174 women) who were admitted to the Diabetes Center of the Osaka City University Hospital for the purpose of glycemic control, education, and/or evaluation of diabetic complications between January 2009 and July 2014. T2D was diagnosed based on the criteria of the American Diabetes Association [20]. Subjects with type 1 diabetes and other types of diabetes were excluded from the present study. In our analyses, a smoker was defined as a current smoker or an ex-smoker. The estimated glomerular filtration rate (eGFR) was calculated by using the Japanese eGFR equation [21], and the subjects were divided into the CKD (eGFR < 60 mL/min/1.73 m^2^) or the non-CKD (eGFR ≥ 60 mL/min/1.73 m^2^) group for analyses.

This study was done in accordance with the Declaration of Helsinki (1975, as revised in 2013). The study protocol was approved by the ethics committee of our institution (number 164). All study participants provided written informed consent.

### 2.2. Physical and Laboratory Measurements

Blood pressure (BP) was determined by using the conventional cuff method with an automatic sphygmomanometer after the subjects had rested for at least 15 min. Blood was drawn after an overnight fast, and biochemical parameters were analyzed by means of a standard laboratory method in the Central Clinical Laboratory of the Osaka City University Hospital (Certification #15-0240 by the Japanese Associations of Medical Technologists) [22]. Glycated hemoglobin A1c (HbA1c) was assessed as the National Glycohemoglobin Standardization Program equivalent value (%), which was expressed by adding 0.4 point to the HbA1c (JDS; %) measured by standard laboratory methods and the previous Japanese standard materials [23]. Immunoreactive insulin levels were measured for subjects not receiving insulin therapy (*n* = 243) by electrochemiluminescence immunoassay (cobas 8000(502/602), Roche Diagnostics) in the Central Clinical Laboratory. Homeostasis model assessment of insulin resistance (HOMA-R) was calculated according to the following formula: fasting insulin (*μ*U/mL) × fasting glucose (mg/dL)/405 [24, 25].

Plasma levels of CTRP9 (SER877Hu, Uscn Life Science, Houston, TX, USA) and total adiponectin (commodity code #410614, Otsuka, Tokyo, Japan) were measured by using an enzyme-linked immunosorbent assay following the manufacturer's instructions. The minimum detectable levels of CTRP9 and adiponectin were 1.29 ng/mL and 23.4 pg/mL, respectively. The intra- and interassay coefficients of variation of CTRP9 were <10% and <12%, respectively, whereas those of adiponectin were <10% and <10%, respectively.

### 2.3. Measurements of Carotid IMT with Ultrasonography

Ultrasonography of the common carotid artery (CCA) was performed by using a phase-locked echo-tracking system, which was equipped with a high-resolution real-time 13 MHz linear scanner (Prosound SSD 6500 and F75; Hitachi Aloka Medical, Ltd., Tokyo, Japan), as previously described [22, 26]. The IMT value was determined by using a measurement software (Intimascope; Media Cross Co. Ltd, Tokyo, Japan), as described elsewhere [27]. In brief, images were obtained 20 mm proximal to the origin of the bulb at the far wall of both CCAs. The average value of 416 points in this region and the largest value, including plaque lesions, in the CCA were measured separately. The mean-IMT of the right and left CCA (mean-IMT) and the greatest IMT among the left and right CCA (max-IMT) were used as markers of atherosclerotic changes.

### 2.4. Statistics

Data are expressed as number (%) or median (interquartile range) as appropriate. For comparisons between the non-CKD and CKD groups, *χ* ^2^-test or Wilcoxon rank-sum test was performed, as appropriate. Correlations were examined by using nonparametric Spearman's rank correlation test. Multiple regression analyses were performed to explore the factors associated with carotid IMT after adjustment for age, sex, body mass index (BMI), systolic BP, eGFR, HbA1c level, triglyceride level, high-density lipoprotein (HDL) cholesterol level, low-density lipoprotein (LDL) cholesterol level, treatment with statins, treatment with angiotensin II receptor blockers or angiotensin-converting enzyme inhibitors (ARBs/ACEIs), smoking status, CTRP9 level, and adiponectin level. A* p* value of <0.05 was considered significant. Statistical analyses were performed by using the JMP 10 software (SAS Institute Inc., Cary, NC, USA).

## 3. Results

### 3.1. Clinical Characteristics of the Subjects

The clinical characteristics of the total study population, as well as of the subjects with and without CKD, are shown in Table 1. The median age, duration of diabetes, and BMI of the subjects were 65 years, 11 years, and 25.0 kg/m^2^, respectively. The median eGFR for all subjects was 67.0 mL/min/1.73 m^2^ (range, 5.8–199.9 mL/min/1.73 m^2^). One hundred and sixty-one subjects (38.4%) were categorized into the CKD group and the remaining 258 (61.6%) were categorized into the non-CKD group. The median eGFR was 42.7 and 76.7 mL/min/1.73 m^2^ for the CKD and the non-CKD group, respectively.

As expected, subjects with CKD were older and had a longer duration of diabetes than those without CKD. The systolic BP and serum triglyceride levels were higher, and the HbA1c, serum HDL-cholesterol, and LDL-cholesterol levels were lower in subjects with CKD than in those without CKD. The prevalence of subjects treated with oral hypoglycemic agents such as sulfonylureas, biguanides, and thiazolidinediones was lower, whereas that of subjects treated with insulin was higher, in the CKD group than in the non-CKD group. The CKD group had a higher prevalence of subjects treated with ARBs/ACEIs and statins for hypertension and dyslipidemia, respectively, than the non-CKD group.

### 3.2. Plasma Levels of CTRP9 and Adiponectin and Carotid IMT in Subjects with T2D

**Figure 1:**
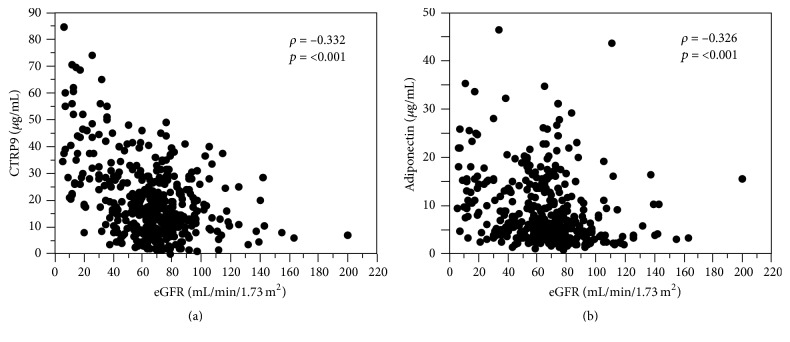
Association of estimated glomerular filtration rate (eGFR) with plasma C1q/tumor necrosis factor-related protein-9 (CTRP9) levels (a) or adiponectin levels (b) in subjects with type 2 diabetes.

The median plasma CTRP9 and adiponectin levels for the total population were 17.4 *µ*g/mL (range, 0.06–84.3 *µ*g/mL) and 6.2 *µ*g/mL (range, 0.85–46.4 *µ*g/mL), respectively. Of note, both plasma CTRP9 and adiponectin levels were inversely correlated with eGFR (Figure 1, Supplementary Tables  1 and  2 in Supplementary Material, available online at http://dx.doi.org/10.1155/2016/8624313), and, accordingly, the plasma levels of CTRP9 and adiponectin were markedly higher in the CKD group than in the non-CKD group (Table 1). No significant correlation between plasma CTRP9 and adiponectin levels was found in either the total population, the non-CKD group, or the CKD group (Supplementary Table 1). In univariate analyses, the plasma CTRP9 levels were positively correlated with age, systolic BP, serum creatinine, and triglycerides and negatively correlated with eGFR, HbA1c, and HDL-cholesterol (Supplementary Table 1). On the other hand, the plasma adiponectin levels were positively correlated with age, diabetes duration, systolic BP, serum creatinine, and HDL-cholesterol and negatively correlated with BMI, eGFR, immunoreactive insulin, HOMA-R, and serum triglyceride levels (Supplementary Table 2).

The median of max-IMT and mean-IMT was 1.08 mm (range, 0.50–2.86) and 0.76 mm (range, 0.34–1.57), respectively. Subjects with CKD had higher max-IMT and mean-IMT than those without CKD (Table 1).

### 3.3. Association between Plasma CTRP9 Levels and Carotid IMT

**Figure 2:**
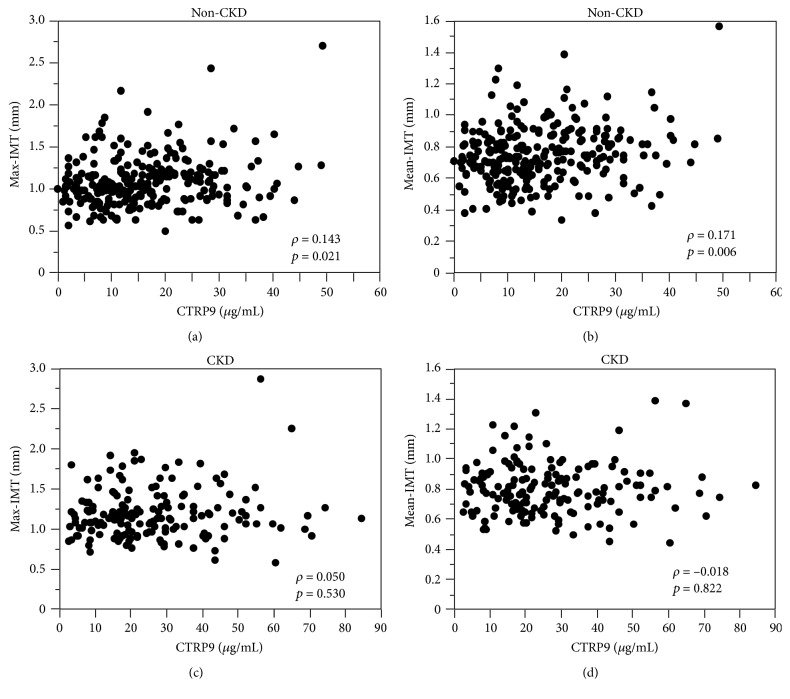
Association of plasma C1q/tumor necrosis factor-related protein-9 (CTRP9) levels with maximum intima-media thickness (IMT) (max-IMT) (a, c) or mean-IMT (b, d) of the common carotid artery in diabetic subjects without chronic kidney disease (CKD) (a, b) or those with CKD (c, d).

We then examined the association of plasma CTRP9 levels with carotid IMT by using univariate analyses in the non-CKD and the CKD groups, separately. In the non-CKD group, plasma CTRP9 levels were positively correlated with max-IMT and mean-IMT (Figures 2(a) and 2(b)). In contrast, neither max-IMT nor mean-IMT was significantly correlated with plasma CTRP9 levels in the CKD group (Figures 2(c) and 2(d)). On the other hand, plasma adiponectin levels were positively correlated with mean-IMT (*ρ* = 0.147, *p* = 0.019), but not with max-IMT (*ρ* = 0.036, *p* = 0.563), in the non-CKD group. Neither max-IMT (*ρ* = 0.039, *p* = 0.627) nor mean-IMT (*ρ* = 0.090, *p* = 0.259) was significantly correlated with plasma adiponectin levels in the CKD group.

### 3.4. Multivariate Analyses of the Determinants for Carotid IMT

Finally, we performed multiple regression analyses to identify an independent association between plasma CTRP9 levels and carotid IMT after adjusting for BMI, plasma adiponectin level, and other potential confounders including age, sex, systolic BP, eGFR, HbA1c, serum triglyceride level, HDL-cholesterol level, LDL-cholesterol level, smoking status, and presence of treatment with statins and ARBs/ACEIs in the non-CKD or the CKD groups, separately. In the non-CKD group, plasma CTRP9 level was found to be an independent determinant of max-IMT (*β* = 0.128, *p* = 0.037) and mean-IMT (*β* = 0.124, *p* = 0.028) (Table 2). Notably, among variables other than CTRP9, the independent determinants were only age for max-IMT and only age and LDL-cholesterol level for mean-IMT in the non-CKD group. On the other hand, no significant association was observed between plasma CTRP9 level and carotid IMT in the CKD group (Table 2).

## 4. Discussion

In the present study, we measured plasma CTRP9 levels in patients with T2D representing a wide range of renal functions and investigated the impact of plasma CTRP9 level on carotid IMT separately in subjects with or without CKD. Plasma CTRP9 levels were positively associated with carotid IMT in diabetic subjects without CKD. Importantly, the association was independent of obesity, plasma adiponectin levels, and other traditional cardiovascular risk factors. The results also revealed that plasma CTRP9 levels were elevated in subjects with CKD compared with those without CKD; however, plasma CTRP9 levels were not significantly associated with carotid IMT in the CKD group.

This study clearly demonstrated that plasma CTRP9 levels were independently and positively associated with carotid IMT in diabetic subjects without CKD. Previous experimental studies in mice consistently demonstrated the beneficial effects of CTRP9 on vascular endothelial function [14], vascular smooth muscle cell proliferation [13], and the profile of inflammatory cytokines in macrophages [28, 29]. Several studies have investigated the association of CTRP9 with cardiovascular risk factors in human subjects [15, 16, 18], whereas only two reports have shown the association of CTRP9 with atherosclerosis [18] or cardiovascular disease [16]. In a study in which 35% of subjects had diabetes [16], the CTRP9 level in serum and epicardial adipose tissue negatively predicted the presence of coronary artery disease. In contrast, a study exclusively of diabetic subjects [18] showed that serum CTRP9 levels were independently and positively associated with arterial stiffness. The present study showed a positive relationship between CTRP9 and carotid IMT, suggesting that the impact of CTRP9 on atherosclerosis or cardiovascular disease differs depending on the characteristics of study population, at least with regard to diabetic status.

Unlike a prior study on subjects with T2D that evaluated subclinical atherosclerosis according to brachial-ankle pulse wave velocity [18], we were able to demonstrate in diabetic subjects a close relationship between plasma CTRP9 level and carotid IMT, the most established surrogate marker for cardiovascular diseases [30]. Moreover, our subjects were older (age, 65 versus 58 years) and had poorer glycemic control (HbA1c, 8.6% versus 7.0%) and lower renal function (eGFR, 76.7 versus 94 mL/min/1.73 m^2^) than those in a prior study [18], even in the non-CKD group. Therefore, this study further suggests a significant impact of CTRP9 on atherosclerosis in patients with T2D who are exposed to a relatively high risk of cardiovascular diseases.

Importantly, the positive relationship between plasma CTRP9 levels and carotid IMT was independent of obesity, plasma adiponectin levels, and other traditional cardiovascular risk factors in the non-CKD group. Considering the inhibitory effect of CTRP9 on vascular smooth muscle cell growth and neointimal formation in mice [13], a detrimental impact of CTRP9 on carotid IMT in this study could be explained by a compensatory response of CTRP9 to the conditions predisposing to the development of atherosclerosis in subjects with T2D. Upregulation of CTRP9 expression in the adipose tissue was observed in 8-week-old* ob/ob* mice relative to age-matched controls [8]. The protein expression of CTRP9 in cardiac tissue was also increased at 4 weeks in mice that received a high-fat diet and decreased thereafter [11]. These findings in mice suggest a compensatory upregulation of CTRP9 in response to obesity. A prior study showing an independent and positive association of serum CTRP9 level with arterial stiffness in human subjects with T2D also indicated a role of CTRP9 as a compensatory factor in the pathogenesis of arterial stiffening [18]. Our research group previously reported that plasma adiponectin levels were inversely associated with carotid arterial stiffness in nondiabetic but not in diabetic subjects [31]. No antiatherogenic effect of adiponectin with carotid IMT was observed in the present study or elsewhere [32, 33]. Taking these results together, a loss of antiatherogenic effect of adiponectin in diabetic condition could be one of the explanations for the compensatory response of CTRP9 to advanced atherosclerosis in patients with T2D. This study also suggests a possibility of a direct pathophysiological link between CTRP9 and atherogenesis in patients with T2D; however, this needs to be confirmed with future studies.

In this study, subjects with CKD exhibited higher plasma CTRP9 levels than those without CKD. Previous studies investigating CTRP9 levels in the circulation of human subjects excluded those with renal dysfunction [15, 16] or targeted T2D subjects with normal renal function [18]. This study is the first to demonstrate elevated plasma CTRP9 levels in human subjects with renal dysfunction compared with those without renal dysfunction. The plasma levels of adiponectin, which belongs to the C1q family and has high homology to CTRP9, were also elevated in subjects with CKD and inversely correlated with eGFR in the present study. The plasma levels of the other isoform of CTRP, CTRP3, were also associated with eGFR in human subjects including those with T2D [34]. Although the elevated adiponectin level in patients with CKD is reported to play a protective role against atherosclerosis and cardiovascular diseases [35, 36], it is currently unknown whether the elevated CTRP9 levels in subjects with CKD play a role or are merely due to reduced renal excretion. In the present study, the plasma CTRP9 levels were associated with systolic hypertension, renal dysfunction, and metabolic dysregulation (e.g., obesity, high triglyceride level, and low HDL-cholesterol level) in the CKD group, whereas no parameter other than age and systolic BP was correlated with CTRP9 levels in the non-CKD group (Supplementary Table 1). These observations may indicate an association of CTRP9 with obesity-related metabolic dysregulation in diabetic subjects with CKD; however, its functional significance needs to be confirmed in further studies.

In contrast to diabetic subjects without CKD, those with CKD exhibited no significant association between plasma CTRP9 levels and carotid IMT. It is well recognized that individuals with CKD are at a higher risk for cardiovascular mortality than those with preserved renal function [19]. In patients with CKD, not only a clustering of several traditional risk factors, but also nontraditional risk factors such as anemia, hyperphosphatemia, hyperhomocysteinemia, and inflammation play a role in cardiovascular damage [37]. Indeed, in the multivariate analyses (Table 2), the explanatory factors including traditional cardiovascular risk factors along with plasma CTRP9 and adiponectin levels did not significantly explain max-IMT (*R* ^2^ = 0.088, *p* = 0.515) or only minimally explained mean-IMT (*R* ^2^ = 0.160, *p* = 0.032) in the CKD group. Therefore, it is possible to speculate that nontraditional factors related to CKD, although not evaluated in this study, attenuated the relationship between CTRP9 and carotid IMT in subjects with CKD.

The concentrations of circulating CTRP9 in this study population were found to be different from those in the prior studies [15, 17, 18], even with the same assay kits. However, it needs to be mentioned that the CTRP9 levels were nearly one hundred times different among the prior studies (mean 4.69 ng/mL [18], 115.3 ng/mL [17], and 415 ng/mL [15]). Moreover, CTRP9 concentrations obtained by the other assay kits were quite different between the assays (96 pg/mL [16] and 5.0 ng/mL [38]). Based on these facts, we should be cautious in comparing the absolute values between studies.

This study has several other limitations. First, we examined only CTRP9 based on its high similarity to adiponectin and the abundance in experimental evidence of its vascular effects [10–14]. Since CTRP1 [39] and CTRP3 [34, 40] were also indicated to be linked to atherosclerosis, the other members of the CTRP family need to be examined in the future. Second, because this was a cross-sectional study, a causal relationship between CTRP9 and carotid atherosclerosis could not be confirmed. Third, our subjects were receiving antihypertensive drugs and statins, which could have affected arterial thickness and its related risk factors. To minimize the effects of these drugs, we adjusted for the presence of these treatments in the multivariate analyses. Fourth, no healthy controls were included in this study, and we could not confirm how diabetic status affects plasma CTRP9 level or its association with carotid IMT. Finally, because our subjects with T2D were hospitalized in a university hospital and were largely without adequate glycemic control, the current results cannot be generalized to the entire population of patients with T2D.

## 5. Conclusions

This study clearly shows that plasma CTRP9 levels are independently and positively associated with carotid IMT in diabetic patients without CKD, but not in those with CKD. Our data indicate an important link between CTRP9 and carotid arterial wall thickness, a powerful predictor of cardiovascular diseases, in patients with T2D. This study further proposes that plasma CTRP9 level is a potential biomarker of atherosclerosis in T2D patients without renal complications. Further longitudinal studies are required to clarify whether plasma CTRP9 levels are predictive of the progression of atherosclerosis in patients with T2D who are at a high risk of cardiovascular diseases.

## Supplementary Material

Supplemental table 1 shows correlation between plasma CTRP9 levels and clinical variables in all subjects with type 2 diabetes (n=419) as well as in subgroups with (CKD group, n = 161) and without CKD (non-CKD group, n=258). Supplemental table 2 shows Correlation between plasma adiponectin levels and clinical variables in all subjects with type 2 diabetes (n=419) as well as in subgroups with (CKD group, n = 161) and without CKD (non-CKD group, n=258).

## Figures and Tables

**Table 1:** Clinical characteristics, plasma CTRP9 levels, plasma adiponectin levels, and carotid IMT in all subjects with type 2 diabetes as well as in subgroups with and without CKD.

	All subjects	Non-CKD	CKD	*p*
*N* (male %)	419 (58.5)	258 (54.3)	161 (65.2)	0.027
Age (years)	65 [55–71]	62 [52–68]	68 [62–74]	<0.001
Duration of diabetes (years)	11 [5–20]	9 [2–17]	16 [10–22]	<0.001
BMI (kg/m^2^)	25.0 [22.0–27.9]	25.1 [21.8–28.2]	24.6 [22.3–27.6]	0.708
Systolic BP (mmHg)	128 [116–143]	124 [113–137]	136 [121–150]	<0.001
Diastolic BP (mmHg)	73 [67–80]	73 [67–79]	74 [67–82]	0.448
Smoker *n* (%)	200 (47.7)	118 (45.7)	82 (50.9)	0.030
Antihyperglycemic agents *n* (%)				
None	43 (10.3)	27 (10.5)	16 (9.9)	0.863
Sulfonylureas	152 (36.3)	107 (41.5)	45 (28.0)	0.005
Biguanides	133 (31.7)	106 (41.1)	27 (16.8)	<0.001
DPP-4 inhibitors	117 (27.9)	75 (29.1)	42 (26.1)	0.508
Thiazolidinediones	48 (11.5)	37 (14.3)	11 (6.8)	0.019
Insulin ± OHA	178 (42.5)	88 (34.1)	90 (56.0)	<0.001
Statin *n* (%)	180 (43.0)	99 (38.4)	81 (50.3)	0.016
ARB/ACEI *n* (%)	169 (40.3)	87 (33.7)	82 (50.9)	<0.001
Fasting glucose (mg/dL)	119 [102–145]	120 [106–147]	117 [95–142]	0.080
HbA1c (%)	8.4 [7.3–9.7]	8.6 [7.6–10.0]	8.2 [7.0–9.4]	0.003
Immunoreactive insulin (*μ*U/mL)^†^	6.7 [4.6–10.1]	6.7 [4.5–10.3]	7.0 [4.6–9.5]	0.876
HOMA-R^†^	2.02 [1.32–2.93]	2.10 [1.33–2.99]	1.85 [1.28–2.61]	0.285
Serum creatinine (mg/dL)	0.81 [0.66–1.08]	0.70 [0.60–0.81]	1.27 [1.02–1.87]	<0.001
eGFR (mL/min/1.73 m^2^)	67.0 [49.3–79.0]	76.7 [68.8–87.3]	42.7 [25.9–53.8]	<0.001
Triglycerides (mg/dL)	116 [90–153]	112 [83–145]	124 [94–176]	0.006
HDL-cholesterol (mg/dL)	41 [36–50]	42 [37–52]	40 [35–47]	0.019
LDL-cholesterol (mg/dL)	106 [84–133]	108 [88–135]	99 [79–128]	0.012
Adiponectin (*μ*g/mL)	6.2 [3.8–12.0]	5.2 [3.5–9.4]	8.4 [4.9–14.7]	<0.001
CTRP9 (*μ*g/mL)	17.4 [9.4–27.8]	13.9 [8.2–22.6]	22.5 [14.9–37.5]	<0.001
Max-IMT (mm)	1.08 [0.92–1.25]	1.05 [0.89–1.20]	1.14 [0.97–1.33]	<0.001
Mean-IMT (mm)	0.76 [0.66–0.87]	0.74 [0.64–0.86]	0.79 [0.68–0.90]	0.005

Data are expressed as median [interquartile range] or *n* (%), as appropriate. *p*  values were determined by using Wilcoxon rank-sum test or *χ* ^2^-test, as appropriate, for comparison between the CKD and non-CKD groups. †, *N* = 243 for all subjects, *n* = 171 for the non-CKD group, and *n* = 72 for the CKD group not receiving insulin therapy. CKD, chronic kidney disease; BMI, body mass index; BP, blood pressure; smoker, prevalence of current or past smokers; DPP, dipeptidyl peptidase; OHA, oral antihyperglycemic agent; statin, prevalence of subjects treated with stains; ARB/ACEI, prevalence of subjects treated with angiotensin II receptor antagonists or ACE inhibitors; HbA1c, glycated hemoglobin A1c; HOMA-R, homeostasis model assessment of insulin resistance; eGFR, estimated glomerular filtration rate; HDL, high-density lipoprotein; LDL, low-density lipoprotein; CTRP, C1q/TNF-related protein; IMT, intima-media thickness.

**Table 2:** Factors independently associated with carotid atherosclerosis in subgroups with and without CKD.

	Max-IMT		Mean-IMT	
	Non-CKD	CKD	Non-CKD	CKD
Age (years)	0.439^*∗∗*^	0.101	0.540^*∗∗*^	0.208^*∗*^
Sex (male = 1, female = 0)	0.046	0.015	0.111	0.114
BMI (kg/m^2^)	−0.051	−0.060	−0.077	−0.228^*∗*^
Systolic BP (mmHg)	0.064	0.110	0.061	0.163
eGFR (mL/min/1.73 m^2^)	0.039	0.097	0.019	0.121
HbA1c (%)	0.025	−0.019	0.014	0.003
Triglycerides (mg/dL)	−0.092	0.054	−0.047	0.066
HDL-cholesterol (mg/dL)	−0.070	−0.118	−0.042	−0.122
LDL-cholesterol (mg/dL)	0.112	0.001	0.146^*∗*^	0.039
ARB/ACEI (yes = 1)	−0.080	0.027	−0.012	−0.074
Statin (yes = 1)	−0.002	−0.194^*∗*^	0.025	−0.066
Smoker (yes = 1)	0.021	0.004	0.036	0.017
Adiponectin (*μ*g/mL)	−0.109	0.013	−0.073	0.004
CTRP9 (*μ*g/mL)	0.128^*∗*^	0.097	0.124^*∗*^	0.078

*R* ^2^	0.234^*∗∗*^	0.087	0.354^*∗∗*^	0.160^*∗*^

The table shows the results of four multiple regression analyses in subjects with type 2 diabetes with CKD and those without CKD. The dependent variables were max-IMT (the left two columns) and mean-IMT (the right two columns). Values are standardized regression coefficients (*β*). *R* ^2^, coefficient of determination; *∗*,  *p* < 0.05; *∗∗*,  *p* < 0.01. BMI, body mass index; BP, blood pressure; eGFR, estimated glomerular filtration rate; HbA1c, glycated hemoglobin A1c; HDL, high-density lipoprotein; LDL, low-density lipoprotein; ARB/ACEI, prevalence of subjects treated with angiotensin II receptor antagonists or ACE inhibitors; statin, prevalence of subjects treated with stains; smoker, prevalence of current and past smokers; CTRP, C1q/TNF-related protein.
