# Supplementary material for: Plasma C1q/TNF-Related Protein-9 Levels Are Associated with Atherosclerosis in Patients with Type 2 Diabetes without Renal Dysfunction
Source: J Diabetes Res. 2016 Dec 14;2016:8624313. doi: 10.1155/2016/8624313 (PMC5192323; doi:10.1155/2016/8624313)
Supplement: Supplementary file 1 — Supplemental table 1 shows correlation between plasma CTRP9 levels and clinical variables in all subjects with type 2 diabetes (n=419) as well as in subgroups with (CKD group, n = 161) and without CKD (non-CKD group, n=258). Supplemental table 2 shows Correlation between plasma adiponectin levels and clinical variables in all subjects with type 2 diabetes (n=419) as well as in subgroups with (CKD group, n = 161) and without CKD (non-CKD group, n=258). [file 8624313.f1.docx]

**Supplemental Table 1. Correlation between plasma CTRP9 levels and clinical variables in all subjects with type 2 diabetes as well as in subgroups with and without CKD**

|  | **All subjects** | | **Non-CKD** | | **CKD** | |
| --- | --- | --- | --- | --- | --- | --- |
|  | ***ρ*** | ***p*** | ***ρ*** | ***p*** | ***ρ*** | ***p*** |
| Age | 0.135 | 0.006 | 0.127 | 0.042 | –0.114 | 0.149 |
| Duration of diabetes | 0.063 | 0.204 | –0.043 | 0.495 | –0.028 | 0.729 |
| BMI | 0.099 | 0.043 | 0.088 | 0.159 | 0.156 | 0.048 |
| Systolic BP | 0.261 | <0.001 | 0.152 | 0.016 | 0.261 | 0.001 |
| Diastolic BP | 0.101 | 0.042 | 0.110 | 0.080 | 0.090 | 0.267 |
| Serum creatinine | 0.260 | <0.001 | –0.150 | 0.016 | 0.433 | <0.001 |
| eGFR | –0.332 | <0.001 | 0.027 | 0.666 | –0.501 | <0.001 |
| Fasting glucose | 0.038 | 0.435 | 0.079 | 0.204 | 0.059 | 0.456 |
| HbA1c | –0.117 | 0.017 | –0.089 | 0.156 | –0.067 | 0.398 |
| Immunoreactive insulin^†^ | 0.038 | 0.568 | 0.055 | 0.481 | 0.001 | 0.993 |
| HOMA-R^†^ | 0.013 | 0.848 | 0.078 | 0.323 | –0.077 | 0.543 |
| Triglycerides | 0.236 | <0.001 | 0.100 | 0.109 | 0.372 | <0.001 |
| HDL-cholesterol | –0.121 | 0.013 | 0.004 | 0.947 | –0.248 | 0.002 |
| LDL-cholesterol | –0.009 | 0.858 | 0.037 | 0.556 | 0.026 | 0.744 |
| Adiponectin | 0.093 | 0.056 | –0.035 | 0.577 | 0.125 | 0.113 |

*ρ*, Spearman’s rank correlation coefficient. †, N=243 for all subjects, n=171 for the non-CKD group, and n=72 for the CKD group not receiving insulin therapy. Abbreviations: CTRP, C1q/TNF-related protein; CKD, chronic kidney disease; BMI, body mass index; BP, blood pressure; eGFR, estimated glomerular filtration rate; HbA1c, glycated hemoglobin A1c; HOMA-R, homeostasis model assessment of insulin resistance; HDL, high-density lipoprotein; LDL, low-density lipoprotein.

**Supplemental Table 2. Correlation between plasma adiponectin levels and clinical variables in all subjects with type 2 diabetes as well as in subgroups with and without CKD**

|  | **All subjects** | | **Non-CKD** | | **CKD** | |
| --- | --- | --- | --- | --- | --- | --- |
|  | ***ρ*** | ***p*** | ***ρ*** | ***p*** | ***ρ*** | ***p*** |
| Age | 0.303 | <0.001 | 0.316 | <0.001 | 0.152 | 0.055 |
| Duration of diabetes | 0.319 | <0.001 | 0.280 | <0.001 | 0.215 | 0.007 |
| BMI | –0.280 | <0.001 | –0.285 | <0.001 | –0.277 | <0.001 |
| Systolic BP | 0.220 | <0.001 | 0.123 | 0.050 | 0.250 | 0.002 |
| Diastolic BP | –0.003 | 0.950 | –0.062 | 0.326 | 0.053 | 0.513 |
| Serum creatinine | 0.213 | <0.001 | –0.014 | 0.829 | 0.267 | 0.001 |
| eGFR | –0.326 | <0.001 | –0.234 | <0.001 | –0.319 | <0.001 |
| Fasting glucose | –0.057 | 0.242 | 0.011 | 0.864 | –0.121 | 0.128 |
| HbA1c | –0.080 | 0.104 | 0.040 | 0.524 | –0.218 | 0.006 |
| Immunoreactive insulin^†^ | –0.346 | <0.001 | –0.330 | <0.001 | –0.408 | <0.001 |
| HOMA-R^†^ | –0.333 | <0.001 | –0.256 | <0.001 | –0.445 | <0.001 |
| Triglycerides | –0.239 | <0.001 | –0.296 | <0.001 | –0.254 | 0.001 |
| HDL-cholesterol | 0.337 | <0.001 | 0.441 | <0.001 | 0.257 | 0.001 |
| LDL-cholesterol | 0.042 | 0.387 | 0.012 | 0.848 | 0.148 | 0.061 |

*ρ*, Spearman’s rank correlation coefficient. †, N = 243 for all subjects, n = 171 for the non-CKD group, and n = 72 for the CKD group not receiving insulin therapy. Abbreviations: CKD, chronic kidney disease; BMI, body mass index; BP, blood pressure; eGFR, estimated glomerular filtration rate; HbA1c, glycated hemoglobin A1c; HOMA-R, homeostasis model assessment of insulin resistance; HDL, high-density lipoprotein; LDL, low-density lipoprotein.
